# Age is a determinant factor in the susceptibility of domestic ducks to H5 clade 2.3.2.1c and 2.3.4.4e high pathogenicity avian influenza viruses

**DOI:** 10.3389/fvets.2023.1207289

**Published:** 2023-07-20

**Authors:** Sun-Hak Lee, Jiho Lee, Jin-Yong Noh, Jei-Hyun Jeong, Jun-Beom Kim, Jung-Hoon Kwon, Sungsu Youk, Chang-Seon Song, Dong-Hun Lee

**Affiliations:** ^1^Avian Disease Laboratory, College of Veterinary Medicine, Konkuk University, Seoul, Republic of Korea; ^2^KHAV Co., Ltd., Seoul, Republic of Korea; ^3^Laboratory of Veterinary Microbiology, College of Veterinary Medicine, Kyungpook National University, Daegu, Republic of Korea; ^4^Department of Microbiology, College of Medicine, Chungbuk National University, Cheongju-si, Republic of Korea; ^5^Wildlife Health Laboratory, College of Veterinary Medicine, Konkuk University, Seoul, Republic of Korea

**Keywords:** high pathogenicity avian influenza, clade 2.3.2.1c H5N1, clade 2.3.4.4e H5N6, age-related pathogenicity, duck

## Abstract

High pathogenicity avian influenza (HPAI) is a viral disease with devastating consequences for the poultry industry worldwide. Domestic ducks are a major source of HPAI viruses in many Eurasian countries. The infectivity and pathogenicity of HPAI viruses in ducks vary depending on host and viral factors. To assess the factors influencing the infectivity and pathogenicity of HPAI viruses in ducks, we compared the pathobiology of two HPAI viruses (H5N1 clade 2.3.2.1c and H5N6 clade 2.3.4.4e) in 5- and 25-week-old ducks. Both HPAI viruses caused mortality in a dose-dependent manner (10^4^, 10^6^, and 10^8^ EID_50_) in young ducks. By contrast, adult ducks were infected but exhibited no mortality due to either virus. Viral excretion was higher in young ducks than in adults, regardless of the HPAI strain. These findings demonstrate the age-dependent mortality of clade 2.3.2.1c and clade 2.3.4.4e H5 HPAI viruses in ducks.

## 1. Introduction

Avian influenza viruses (AIVs), members of the genus Influenza virus A of the family Orthomyxoviridae, are divided into subtypes based on the surface glycoproteins hemagglutinin (HA, H1-H16) and neuraminidase (NA, N1–N9) (1). The natural reservoirs of most AIVs are wild aquatic birds, especially those of the orders Anseriformes (ducks, geese, and swans) and Charadriiformes (gulls, terns, and waders). They play a major role in the evolution, maintenance, and dissemination of AIVs (2). Most AIVs in natural hosts are low-pathogenic avian influenza (LPAI) viruses that cause little or no disease in natural hosts and Gallinaceous poultry (3). However, novel high pathogenicity avian influenza (HPAI) viruses arise following the adaptation of the H5 and H7 subtypes in domestic poultry and cause significant illness or death (4). Since the detection of A/Goose/Guangdong/1/1996(H5N1) (Gs/GD) in domestic poultry in southern China, the descendant viruses have evolved into 10 genetically distinct hemagglutinin (HA) clades (0–9) (5). Along with prolonged circulation in poultry, the predominant subclades of clade 2 H5 viruses were replaced by an antigenically distinct subclade, followed by 2.2 (6) and 2.3.2.1 (7), and further evolved into three subclades, 2.3.2.1a, 2.3.2.1b, and 2.3.2.1c (8), mainly in China and Southeast Asia. Various HPAI subtypes bearing the genetic backbone of the Gs/GD lineage H5 clade 2.3.4.4 have been identified in domestic ducks since 2008 and have subsequently evolved into different subclades (9). Among them, clades 2.3.2.1c H5N1 and 2.3.4.4b H5NX HPAI spread intercontinentally via wild migratory birds, causing earlier intercontinental waves (waves 2 and 3a) and subsequent waves (waves 3b and 4), respectively (10).

Domestic ducks play a substantial role in the evolution, maintenance, and spread of Gs/GD HPAI. For example, novel genotypes of HPAI H5N1 in Bangladesh and HPAI H5N5 and H5N8 in China have been reported in domestic ducks, highlighting the role of domestic ducks as reassortment vessels for creating new genotypes of influenza viruses (11). Some studies have emphasized that domestic duck populations and transport could affect the prevalence and distribution of HPAI viruses, particularly in countries where ducks are the main food source. During the HPAI H5N1 outbreaks from 2007 to 2009 in South and Southeast Asia, the population of domestic ducks was the main factor delineating areas at risk of HPAI H5N1 spreading in domestic poultry (12). For the novel introduction of clade 2.3.4.4 H5N8 viruses that occurred in South Korea in 2014, wild waterfowl migration and domestic duck density have shaped the epidemiology of H5N8 viruses (13). Under the unique fattening duck production system known as ‘foie gras’ in southwest France, the trade-related transport of fattening ducks contributed to the 2016–2017 epizootic of HPAI H5N8 in France (14).

The clinical signs and mortality of HPAI viruses vary. In ducks, depending on various factors, including viral strains and pre-immune status. Some strains of HPAI viruses induce subclinical infection that can facilitate the spread and persistence of HPAI viruses (15–19) Mortality among ducks naturally infected with the HPAI virus was first reported in Italy (20), and Asian-origin H5N1 viruses have caused mortality in wild and domestic ducks (21). In contrast, different pathogenicities in ducks have been observed between distinct strains of HPAI viruses in several previous studies. The Hong Kong H5N1 HPAI isolates in 1997 caused limited pathogenicity in ducks (22), but the 2002 HPAI isolates caused increased mortality and systemic infections in ducks (23). Comparison studies showed differences in pathogenicity between two H5N1 HPAI isolates from Egypt in 2007 and 2008 (24) and two H5N6 HPAI isolates from Korea in 2016. However, host factors, especially age at infection, which possibly affects pathogenicity, have not been fully understood in recent clades of the HPAI virus. This study aimed to assess the factors influencing the infectivity and pathogenicity of HPAI viruses in ducks. We compared the pathobiology of two HPAI viruses (H5N1 clade 2.3.2.1c and H5N6 clade 2.3.4.4e) in 5- and 25-week-old ducks.

## 2. Materials and methods

### 2.1. Viruses

We used two HPAI viruses, A/duck/Korea/ES2/2016 (H5N6, ES2) clade 2.3.4.4e and A/chicken/Vietnam/NCVD-KA435/2013 (H5N1, KA435) clade 2.3.2.1c, for experimental infection. The viruses were kindly provided by the Animal and Plant Quarantine Agency of Korea. Viruses were inoculated into 9–11 days old specific-pathogen-free embryonated chicken eggs, and allantoic fluids were harvested after 2–3 days of incubation at 37°C. The virus was aliquoted and stored in a −70°C deep freezer for further experiments. The titration endpoints for each virus were calculated using standard methods (25).

### 2.2. Animals

We used 37 five-week-old domestic ducks and 40 25-week-old domestic ducks obtained from the Moran Food & Breeding Company (Eum-Seong, Republic of Korea). All oropharyngeal and cloacal swabs were negative for influenza virus infection based on the real-time reverse transcription-polymerase chain reaction (rRT-PCR) (25). Before the viral challenge, all ducks were confirmed to be seronegative for anti-AIV antibodies using a commercial emzyme-linked immunosorbent assay (ELISA) kit (Bionote, Korea). All ducks used in this study were housed in self-contained isolation cages in a controlled environment at the ABSL-3 facility at Konkuk University to maintain biosafety and biosecurity barriers. All animal procedures were reviewed, approved, and supervised by the Institutional Animal Care and Use Committee (IACUC) (no. KU1840, KU18193), and the Institutional Biosafety Committee (No. KUIBC-2018-10, KUIBC-2019-05) at Konkuk University.

### 2.3. Experimental design

Five-week-old (*n* = 27) and 25-week-old (*n* = 36) ducks were divided into two groups. Each group was inoculated with the ES2 virus (*n* = 13 for younger ducks and *n* = 14 for older ducks) or the KA435 virus (*n* = 18 for each age group). Six 5-week-old and eight 25-week-old ducks were used as negative controls. To evaluate the mean bird infectious dose (BID_50_) and mean bird lethal dose (BLD_50_), we divided each age group into three groups (4–6 ducks). Ducks were inoculated intranasally with 10^4^, 10^6^, or 10^8^ 50% egg infective doses (EID_50_) of the viruses, hereafter referred to as low, medium, and high doses, respectively. Ducks were observed daily for clinical signs and mortality after the challenge for 14 days. To detect and quantify viral shedding, oropharyngeal and cloacal swabs were collected at 3, 5, 7, 10, and 14 day-post-challenge (dpc) and submerged in 1.5 ml PBS. Sera were collected from the birds 14 d after infection to verify seroconversion. A commercial competitive ELISA kit (Bionote, Korea) was used to detect anti-AIV antibodies targeting nucleocapsid protein (NP) according to the manufacturer’s instructions. Ducks were considered infected if they were seroconverted by 14 dpc or had detectable viruses, along with clinical signs and mortality.

### 2.4. Clinical scoring

Clinical scores were determined by applying the IVPI scoring system on ducks (26, 27). Ducks were observed daily for 14 days post infection. Birds were scored 0 if healthy, 1 if sick, 2 if severely sick, and 3 if dead. Birds were considered ‘sick’ if one of the following signs was observed and considered ‘severely sick’ if more than one of the following signs were observed: respiratory involvement, depression, diarrhea, cyanosis of the feet or mucosa, edema of face or head, and nervous signs. Clinical scores were calculated per group with an observation period of 10 days. When ducks were too sick or could not be urged to move, they were killed humanely and scored as dead.

### 2.5. Viral RNA quantification

Viral RNA from oropharyngeal and cloacal swabs was extracted from 200 μL of the supernatant using the MagNA Pure 96 extraction system (Roche, Manheim, Germany) according to the manufacturer’s instructions. The extracted RNA was quantified by real-time reverse transcription-polymerase chain reaction using previously described protocols (28). The Ct values were converted into infectious units equivalent to EID_50_/ml using a standard curve.

### 2.6. Statistical analysis

The Kaplan–Meier survival curve was constructed, and the Mantel–Cox log-rank test was used to compare the survival curves between the two age groups. An unpaired *t*-test was applied for normally distributed data; otherwise, the Mann–Whitney U test was used. All statistical analyses were performed using GraphPad Prism version 8.2.1 (GraphPad Software Inc., CA, USA). Statistical significance was set at *p* ≤ 0.05.

## 3. Results

### 3.1. Infectivity, mortality, and clinical signs

**Figure 1:**
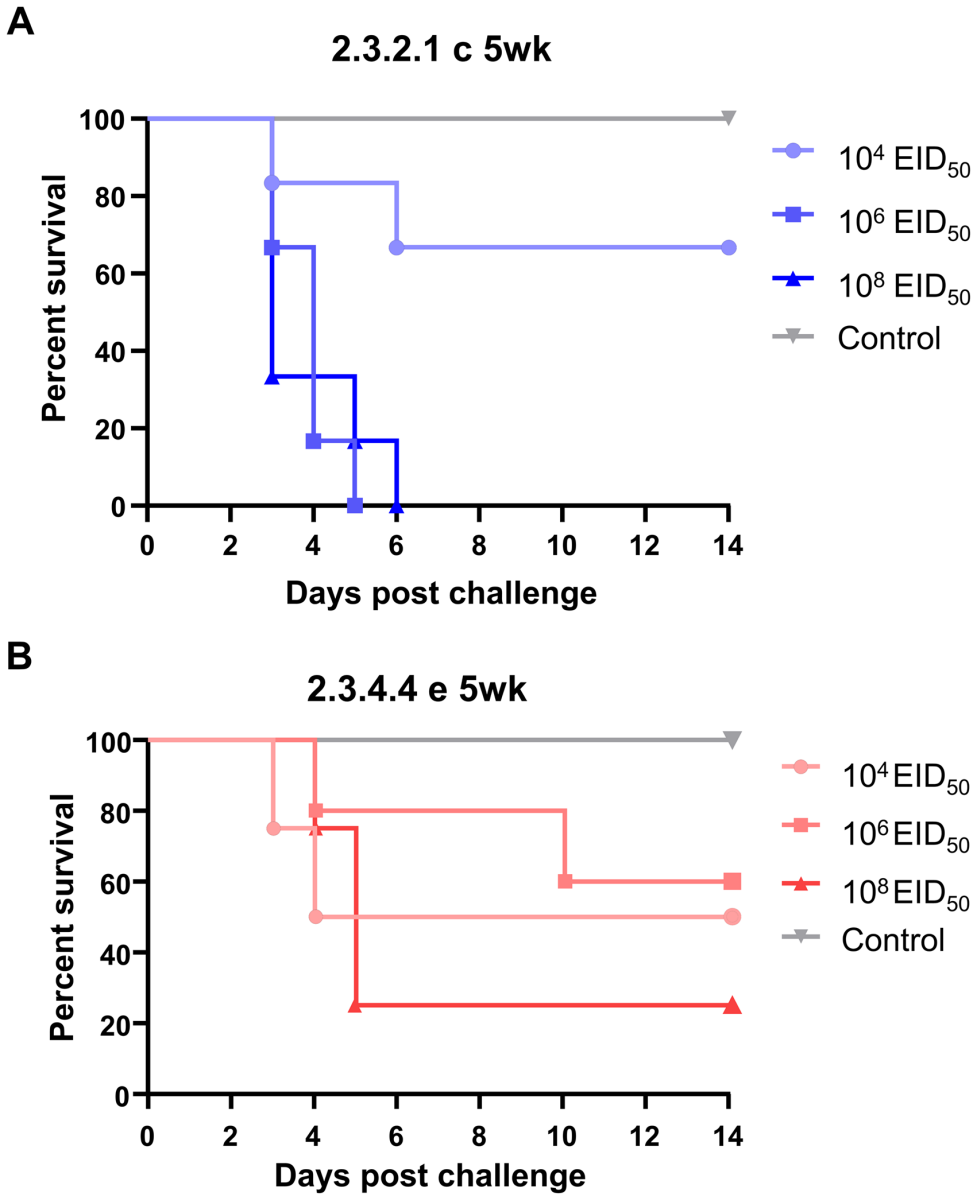
Survival curves of 5-week-old ducks challenged with low, medium, or high doses of H5N1 2.3.2.1c HPAI virus **(A)** and H5N6 2.3.4.4e HPAI virus **(B)**. The Kaplan–Meier survival curve was constructed using Prism 8.2.1, with data from the 63 subjects’ mortality records. The Mantel–Cox log-rank test evaluated significance (*p* < 0.05).

None of the negative control ducks of either age exhibited viral shedding, seroconversion, or clinical signs (Figure 1). In the groups inoculated with the KA435 virus, all 5-week-old ducks died, except for four out of six ducks challenged with the low dose. None of the 5-week-old ducks had anti-AIV antibodies. All 5-week-old ducks in the medium- and high-dose challenge groups died within 6 days, with a mean death time (MDTs) of 3.8 days (Table 1). For the group infected with a low dose, two out of six ducks died within 6 days, and the MDT was 4 days. Ducks died before 4dpc did not show any clinical signs, while torticollis and incoordination started to appear after 4dpc on ducks that succumbed to death. The BID_50_ and BLD_50_ were 10^4.5^EID_50_. In contrast, none of the 25-week-old ducks challenged with the KA435 virus died while some ducks showed depression and respiratory involvement (Supplementary Figure 1A). Based on the serologic examination, all 25-week-old ducks challenged with the high-dose virus were seroconverted, followed by four out of six ducks in the medium-dose group and one out of six ducks in the low dose challenge group, resulting in a BID_50_ dose of 10^5.27^EID_50_.

**Table 1:** Mortality, morbidity, mean death time, BID_50_, and BLD_50_ of H5N1 clade 2.3.2.1C and H5N6 clade 2.3.4.4e in 5-week-old ducks and 25-week-old ducks.

Age	Virus strain	Dose^a^	Morbidity^b^	Mortality^c^	MDT^d^	BID_50_^e^	BLD_50_^f^
5-week-old	2.3.2.1c A/chicken/Vietnam/ NCVD-KA435/ 2013(H5N1)	High (10^8.0^EID_50_)	6/6	6/6	3.8	10^4.5^EID_50_	10^4.5^EID_50_
		Medium (10^6.0^EID_50_)	6/6	6/6	3.8		
		Low (10^4.0^EID_50_)	2/6	2/6	4		
	2.3.4.4e A/duck/Korea/ES2/ 2016(H5N6)	High (10^8.0^EID_50_)	4/4	3/4	4.7	<10^4.0^EID_50_	10^5.26^EID_50_
		Medium (10^6.0^EID_50_)	5/5	3/5	6.7		
		Low (10^4.0^EID_50_)	3/4	2/4	3.5		
25-week-old	2.3.2.1c A/chicken/Vietnam/ NCVD-KA435/ 2013(H5N1)	High (10^8.0^EID_50_)	6/6	0/6	ND^g^	10^5.27^EID_50_	>10^8.0^EID_50_
		Medium (10^6.0^EID_50_)	4/6	0/6	ND		
		Low (10^4.0^EID_50_)	1/6	0/6	ND		
	2.3.4.4e A/duck/Korea/ES2/ 2016(H5N6)	High (10^8.0^EID_50_)	5/5	0/5	ND	<10^4.0^EID_50_	>10^8.0^EID_50_
		Medium (10^6.0^EID_50_)	4/5	0/5	ND		
		Low (10^4.0^EID_50_)	4/4	0/4	ND		

a Ducks were inoculated intranasally with each dose of the viruses.

b Number of infected ducks confirmed with viral shedding or seroconversion/number of inoculated ducks.

c Number of dead ducks/number of inoculated ducks.

d Mean death time in days.

e 50% Bird infectious dose.

f 50% Bird lethal dose.

g Not detectable.

For the ES2 virus, the BLD_50_ of 5-week-old ducks was 10^5.26^EID_50_, as three out of four ducks (high-dose), three out of five ducks (medium-dose), and two out of four ducks (low dose) died after the challenge. Severe clinical signs, such as incoordination and torticollis, were observed in the four out of eight ducks died from the infection (Supplementary Figure 1B). A few ducks challenged with the ES2 virus survived and seroconverted, resulting in a lower BID_50_ (< 10^4.0^EID_50_) than the BLD_50_. The MDTs of the younger ducks challenged with a high, medium, and low dose was 3.5, 6.7, and 4.7 days, respectively. Consistent with the KA435 virus, none of the 25-week-old ducks died after the challenge with the ES2 virus. No ducks showed incoordination or nervous signs, while six ducks showed depression or mild respiratory involvements. The morbidity rates of the high-, medium-, and low-dose challenge groups were 100% (five out of five ducks), 66.6% (four out of six ducks), and 16.6% (one out of six ducks), respectively, indicating that the BID_50_ of the ES2 virus in 25-week-old ducks was 10^5.27^EID_50_.

The survival curves of different age groups inoculated with the same dose of the virus were compared using the log-rank test (Figure 1). The survival curves of the groups inoculated with high and medium doses of the KA435 virus showed significant differences (*p* = 0.001 and *p* = 0.0014, respectively, Figure 1A; Supplementary Figure 2) in the survival rate between 5-week-old and 25-week-old ducks. Adult ducks that received a high-dose of the ES2 virus were significantly less likely to exhibit mortality than young ducks with the same challenge dose and strain (*p* = 0.0148, Supplementary Figure 2). In addition, the survival curves of groups inoculated with the same doses of KA435 and ES2 viruses were compared (Supplementary Figure 3). These data demonstrated significant differences between the two viruses only at medium doses using the log-rank test (*p* = 0.008, Supplementary Figure 3). Young ducks inoculated with a medium-dose of the KA435 virus had a significantly lower estimate of survival than young ducks inoculated with the same dose of the ES2 virus. No statistically significant difference was observed between the survival curves for the other two doses.

### 3.2. Viral shedding

**Figure 2:**
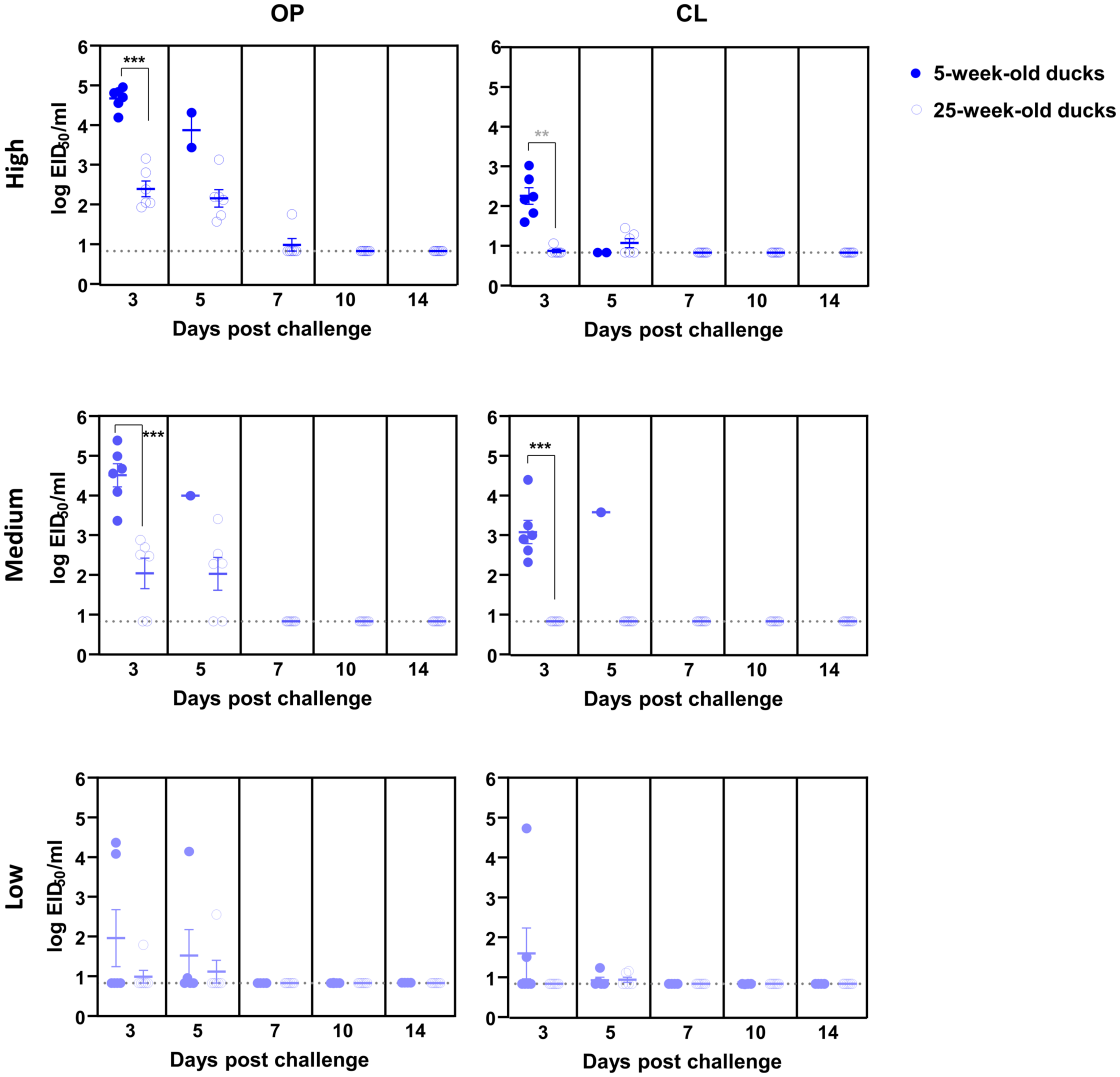
Oropharyngeal (OP) and cloacal (CL) virus shedding in 5- and 25-week-old ducks challenged with low, medium, or high doses of H5N1 2.3.2.1c HPAI virus. The dotted line indicates the detection limit (10^0.8345^ EID_50_ equivalent/0.1 ml). The middle line among circles indicates the mean value and error bars indicate standard deviation. The black asterisks indicate that statistical analyses were conducted using an independent samples *t*-test. The grey asterisks indicated that statistical analyses were conducted using a Mann–Whitney U Test (**p < 0.01; ***p < 0.001).

**Figure 3:**
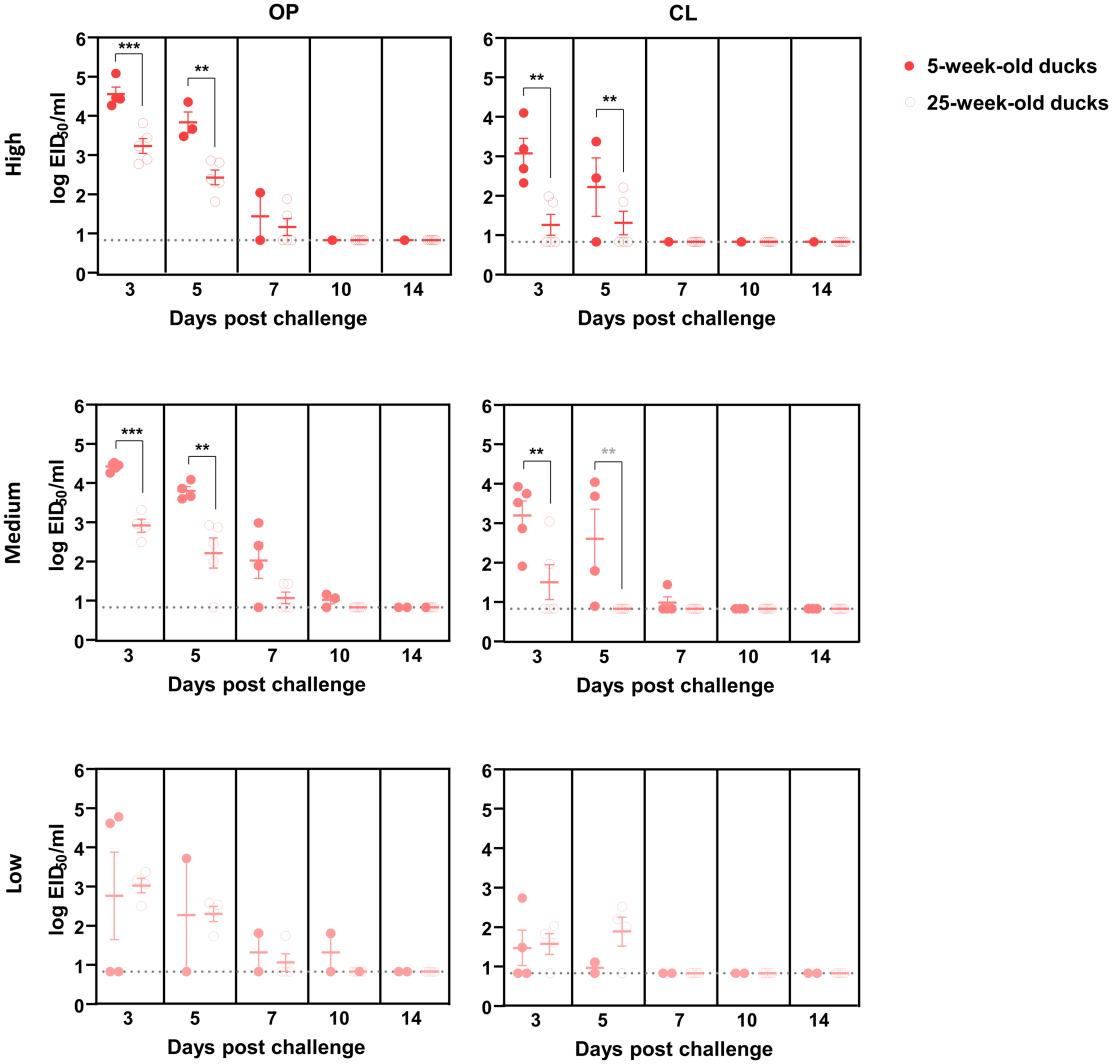
Oropharyngeal (OP) and cloacal (CL) virus shedding in 5- and 25-week-old ducks challenged with low, medium, or high doses of H5N6 2.3.4.4e HPAI virus. The dotted line indicates the detection limit (10^0.8345^ EID_50_ equivalent/0.1 ml). The middle line among circles indicates the mean value and error bars indicate standard deviation. The black asterisks indicated that statistical analyses were conducted using an independent samples *t*-test. The grey asterisks indicated that statistical analyses were conducted using a Mann–Whitney U Test (**p < 0.01; ***p < 0.001).

Oropharyngeal (OP) and cloacal (CL) swabs collected from virus-inoculated ducks at different time points were analyzed using quantitative rRT-PCR to measure viral shedding. The mean viral titer of OP swabs on each swab day was consistently higher than that of CL swabs for both viruses. In general, virus shedding peaked at 3 dpc and then gradually declined, regardless of the dose and virus strain, except for the 25-week-old ducks inoculated with a low dose of ES2. For high- and medium-dose groups inoculated with KA435, 5-week-old ducks shed more virus than 25-week-old ducks in both OP and CL routes at 3 dpc (Figure 2, *p* < 0.001 for OP-high, OP-medium, and CL-medium, and *p* < 0.01 for CL-high). Virus shedding was also higher in 5-week-old ducks challenged with high and medium doses of ES2 at 3 and 5 dpc (Figure 3; *p* < 0.001 for OP-high, medium at 3 dpc, and p < 0.01, for the others). Therefore, for the ducks that were confirmed to be infected, 5-week-old ducks shed significantly higher amounts of the virus *via* the OP and CL routes than 25-week-old ducks, regardless of the virus strain.

## 4. Discussion

Previous natural and experimental infection studies have shown that HPAI-infected wild and domestic waterfowls present no to mild clinical signs (15–19). For example, early Hong Kong H5N1 HPAI isolates from 1997 showed limited pathogenicity in ducks (22). However, the continuous evolution of HPAI viruses has increased their lethality in various bird species, including ducks. Many previous studies have demonstrated that HPAI viruses induce varied pathogenicities in ducks. Unlike the 1997 H5N1 HPAI isolates, the novel 2002 HPAI isolates caused systemic infection in ducks, with high virus titers and pathology in multiple organs, causing neurological dysfunction and death (23). Mortality in wild and domestic ducks is caused by many Asian-origin HPAI H5N1 viruses (21). Recently, mass die-off cases of tufted ducks (*Aythya fuligula*) were reported in the Netherlands (29, 30) and Germany (31) from 2016 to 2017, and high wild bird mortality was observed in coastal and other water-rich areas of the Netherlands between October 2020 and June 2021 (32). In this study, the ES2 virus, which belongs to clade 2.3.4.4e, had a lower BID_50_ and higher BLD_50_ in 5-week-old ducks than KA435, which belongs to clade 2.3.2.1c HPAI clade. Recent studies have reported that the molecular changes associated with the unusual lethality of HPAI viruses in ducks are related to the PA and PB1 genes of the H5N1 virus (33), PA and NS genes of clade 2.3.4.4 H5N6 virus (34), and PB2, NP, and M genes of clade 2.3.4.4 H5N8 virus (35). However, the underlying mechanisms have not been identified.

Our data indicate that younger ducks are more susceptible to both the 2.3.2.1c and 2.3.4.4e challenge viruses. Our findings are consistent with those of other studies by Pantin-Jackwood et al. (36), Jang et al. (37), and Londt et al. (38), in that the younger the host age, the more severe the clinical signs and higher the mortality were observed, while variances existed in the degree of mortality rate and clinical signs in accordance with the strain challenge and age at infection. In other words, some strains of Gs/GD H5 HPAI viruses, such as A/chicken/Hong Kong/220/97 (H5N1), A/Egret/HK/757.2/02 (H5N1), A/Duck/Vietnam/218/05 (H5N1) (36), and A/Waterfowl/Korea/S57/2016 (H5N6) (37), showed age-dependent pathogenicity in ducks. Age-related pathogenicity of HPAI has also been reported in turkeys (39), wild ducks (ruddy ducks, lesser scaups) (40), and humans (41), but not in broiler chickens (42). In a previous study, the BID_50_ and BLD_50_ of the ES2 virus in 2-week-old ducks were 10^3.0^EID_50_ and 10^4.0^ EID_50_, supporting the younger the duck is, the more vulnerable it is to the HPAI virus (34). The challenged ducks exhibited higher viral titers in the OP swabs than in the CL swabs in this study, consistent with previous studies using other HPAI viruses, including H5N1 viruses isolated in 2002 and 2004 (18), and H5N8 and H5N6 viruses isolated in South Korea in 2016 and 2017, respectively (19).

No mortality was observed in 25-week-old ducks, even at a high-dose of inoculation, but excreted viruses, suggesting that adult ducks could play a significant role in the maintenance and spread of HPAI viruses. Also, ducks that showed viral shedding did not show any clinical signs, indicating their role as a silent reservoir of the virus. Innate immunity and receptor distribution are suspected to be factors affecting age-dependent pathogenicity. Studies on Pekin ducks suggest that higher body temperature and upregulation of innate immune-related genes, including IFN-α, retinoic acid-inducible gene I (RIG-I), and IL-6 in spleens, could impact the age-related pathogenicity of several H5N1 HPAI viruses (36). In a pathogenicity study of the H5N6 HPAI virus isolated in South Korea in 2016, cell damage-related genes, such as CIDEA and ND2, and the immune response-related gene NR4A3 were dramatically induced in the lungs of infected 2-week-old ducks compared with those in the lungs of 4-week-old ducks (37). Age-dependent α-2,6 sialic acid expression variations among minor poultry species have been observed in ducks, geese, and turkeys (43). In turkeys, sialic acid receptor patterns change with age, which can result in variations in viral replication and tissue tropism. As poultry species age, the migration of lymphocytes to peripheral mucosa-associated lymphoid tissue (MALT) increases (44), which could lead to mortality in younger ducks and a higher inflammatory response against HPAI viral replication. Based on these studies, it is reasonable to hypothesize that variations in viral pathogenicity may arise from differences in innate immunity, immune response-related gene expression, and receptor expression according to age. Consequently, analyzing these factors in poultry, including ducks of different ages, can provide crucial data for comprehending the responses to viral infections.

Other host and environmental factors can also affect duck pathogenicity. In the case of host factors, as presented in this study, age at infection is a determinant factor for some strains in certain species; consistently, younger groups showed high pathogenicity (18, 36–40). The presentation of the disease also varied by virus strain and duck species, with Muscovy ducks being more vulnerable than Pekin ducks (45), and pre-existing immunity by commercial vaccination (46) or immunosuppressive viral infections (47). Co-infections with other respiratory pathogens could influence the outcome of infection, as seen in previous studies using the duck hepatitis virus (48) and other subtypes of LPAI viruses (49). Infections and pathogenicity could also be influenced by environmental factors, leading to higher virus concentrations and persistence, such as elevated virus levels due to ventilation and longer virus survival under favorable environmental conditions (50). Altogether, the pathogenicity of HPAI viruses depends on many factors, which could raise various patterns of disease, including the diverse onset of infections, clinical signs, and mortality. Further studies should be conducted to investigate the pathogenicity and related factors of recently circulating viruses to understand the mechanisms of the disease.

The pathogenicity of HPAI can be influenced by a range of factors, including viral characteristics and environmental conditions, as well as host physiology and immune response. In this study, we investigated the impact of age on the pathogenicity of two clades of HPAI viruses in ducks, in terms of mortality, infectivity, and level of virus shedding. Our results showed that younger ducks exhibited higher pathogenicity, as evidenced by increased mortality rates and viral shedding, compared to older ducks. The results obtained in this study may help gain insight into age-related differences in the transmission dynamics and disease patterns of viruses. The contrasting survival between younger and older ducks suggests that silent infection and transmission can occur in older ducks, indicating that active surveillance and risk assessment should be carefully implemented in aged ducks in the earlier stages of HPAI outbreaks to prevent them from spreading. In addition, since ducks can host a variety of avian influenza viruses as a natural reservoir species, older ducks with asymptomatic or mild infections can play a role in evolution of HPAIVs, potentially giving rise to new strains with altered pathogenicity or increased zoonotic potential. The precise mechanisms causing the higher virulence in young ducks remain unknown and warrant further investigation.

## Data availability statement

The raw data supporting the conclusions of this article will be made available by the authors, without undue reservation.

## Ethics statement

The animal study was reviewed and approved by Institutional Animal Care and Use Committee, Institutional Biosafety Committee.

## Author contributions

JL, J-YN, J-HJ, and J-BK contributed to conceptualization, methodology, formal analysis, investigation, resources, and data curation. S-HL and JL wrote the first draft of the manuscript. S-HL, JL, J-HK, SY, and D-HL wrote review and editing. Project administration and funding acquisition were performed by JL, J-YN, J-HJ, J-BK, and C-SS. All authors contributed to the article and approved the submitted version.

## Funding

This work was supported by the Korea Institute of Planning and Evaluation for Technology in Food, Agriculture, Forestry (IPET) through the Animal Disease Management Technology Development Program, funded by the Ministry of Agriculture, Food, and Rural Affairs (MAFRA) (grant number: 318032). This paper was supported by Konkuk University in 2022.

## Conflict of interest

J-YN, J-HJ, J-BK and C-SS are employed by KHAV Co., Ltd.

The remaining authors declare that the research was conducted in the absence of any commercial or financial relationships that could be construed as a potential conflict of interest.

## Publisher’s note

All claims expressed in this article are solely those of the authors and do not necessarily represent those of their affiliated organizations, or those of the publisher, the editors and the reviewers. Any product that may be evaluated in this article, or claim that may be made by its manufacturer, is not guaranteed or endorsed by the publisher.
