# Supplementary figures and images for: Age is a determinant factor in the susceptibility of domestic ducks to H5 clade 2.3.2.1c and 2.3.4.4e high pathogenicity avian influenza viruses
Source: Front Vet Sci. 2023 Jul 20;10:1207289. doi: 10.3389/fvets.2023.1207289 (PMC10400362; doi:10.3389/fvets.2023.1207289)

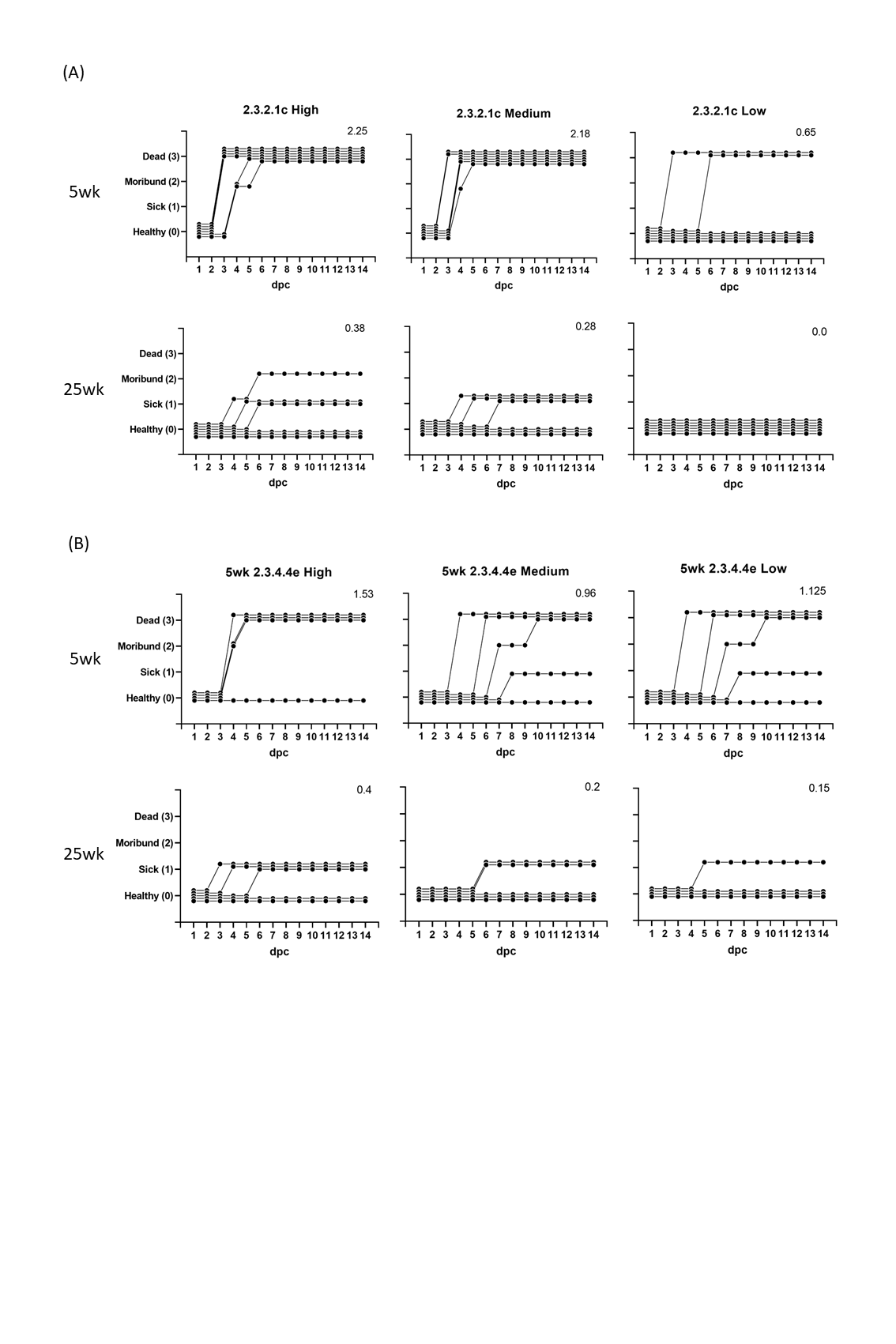

Supplement: Supplementary file 1 [file Image_1.tif]
